# Assessment of Efficiency and Safety of Apatinib in Advanced Bone and Soft Tissue Sarcomas: A Systematic Review and Meta-Analysis

**DOI:** 10.3389/fonc.2021.662318

**Published:** 2021-03-17

**Authors:** Zuoyao Long, Mengquan Huang, Kaituo Liu, Minghui Li, Jing Li, Hongmei Zhang, Zhen Wang, Yajie Lu

**Affiliations:** ^1^ Department of Orthopedics, Xijing Hospital, Air Force Medical University of PLA, Xian, China; ^2^ Department of Burns and Cutaneous Surgery, Xijing Hospital, Air Force Medical University of PLA, Xian, China; ^3^ Department of Oncology, Xijing Hospital, Air Force Medical University of PLA, Xian, China

**Keywords:** apatinib, osteosarcoma, soft tissue sarcoma, advanced, meta-analysis

## Abstract

**Background:**

Previous studies, both *in vitro* and *in vivo*, have established that apatinib has anti-tumor properties. However, insufficient empirical evidence of the efficacy and safety of apatinib has been published for bone and soft tissue sarcoma, the reported results differing widely. Here, we conducted a meta-analysis to assess the efficacy and toxicity of apatinib for the treatment of bone and soft tissue sarcoma.

**Methods:**

Pubmed, Medline, Web of Science, ScienceDirect, Ovid, Embase, Cochrane Library, Scopus, Vip (China), Cnki (China), Wanfang (China), and CBM (China) databases and literature from conferences were searched for studies of apatinib for the treatment of bone and soft tissue sarcomas, published from the inception of each database to Sep 1, 2020, without language restrictions. Primary outcomes were efficacy and toxicity of apatinib for the treatment of bone and soft tissue sarcoma, including treatment response, progression-free survival (PFS), and the incidence of adverse events. After extraction of data and methodological quality evaluation, random or fixed-effects models, as appropriate, were selected to calculate pooled effect estimates using R software (Version 3.4.1).

**Results:**

A total of 21 studies with 827 participants were included in the present meta-analysis. The mean MINORS score was 10.48 ± 1.75 (range: 7-13), indicating evidence of moderate quality. Pooled outcomes indicated that overall response rate (ORR) and disease control rate (DCR) were 23.85% (95% CI: 18.47%-30.21%) and 79.16% (95% CI: 73.78%-83.68%), respectively. Median PFS ranged from 3.5 to 13.1 months, with a mean of 7.08 ± 2.98 months. Furthermore, the rates of PFS (PFR) after 1, 6, and 12 months were 99.31%, 44.90%, and 14.31%, respectively. Drug-related toxicity appears to be common in patients administered apatinib, for which hand-foot syndrome (41.13%), hypertension (36.15%), and fatigue (20.52%) ranked the top three most common adverse events. However, the incidence of grade 3-4 adverse events was relatively low and manageable.

**Conclusions:**

Based on the best evidence currently available, apatinib demonstrates promising clinical efficacy and an acceptable safety profile for the treatment of advanced bone and soft tissue sarcoma, although additional high-quality clinical studies are required to further define its properties and toxicity.

## Introduction

Bone and soft tissue sarcomas (STS) are rare malignant tumors originating from mesenchymal tissues that are present at all ages (1). They comprise more than 50 subtypes with different histological features and clinical behavior (2). Complete tumor resection with a sufficient surgical-margin is the most effective primary therapy for bone sarcoma and STS, alone or in combination with radiotherapy and/or chemotherapy. Although significant improvements in multidisciplinary approaches have prolonged the survival of patients with bone sarcoma or STS, it displays a poor response and unfavorable prognosis for advanced, refractory, metastatic, or relapsed sarcomas (3–5).

Tumor angiogenesis plays a crucial role in the initiation of tumors, and their progression, invasion, and metastasis (6, 7). Antiangiogenic therapies represent a promising anticancer treatment strategy, already extensively used for the treatment of solid tumors, aiming to inhibit tumor angiogenesis by targeting the key signaling pathways in which it is implicated (8–10).

Tumor-derived angiogenic factors have been identified as members of the vascular endothelial growth factor (VEGF) family, participating in the process of tumor angiogenesis *via* interaction with the corresponding receptor (VEGFR), suggesting that disruption of the VEGF/VEGFR signaling pathway could suppress the growth of tumors (11). A number of VEGFR inhibitors (e.g. sorafenib, axitinib, pazopanib, etc.) have been developed following two decades of laboratory research and clinical trials, demonstrating clinical effectiveness in a variety of cancers (12–15). Unfortunately, the anticipated anti-tumorigenic properties have not been observed in advanced bone and soft tissue sarcomas, compared with other malignancies (16, 17).

Apatinib (YN968D1) is a small molecule tyrosine kinase inhibitor (TKI) which selectively inhibits VEGFR2, thereby blocking the VEGF/VEGFR signaling pathway, and inhibiting tumor angiogenesis (18). It was approved by the Chinese FDA in December 2014 for the clinical treatment of advanced gastric cancer based on significant improvement in overall survival (OS) and progression-free survival (PFS) in a phase III clinical trial (19, 20). Over the past few years, apatinib has demonstrated broad-spectrum antitumor effects in numerous trials for multiple malignancies, including lung cancer, breast cancer, and hepatoma, etc. (21–24). Apatinib has also been shown to display anti-angiogenic properties in advanced sarcoma, and its clinical efficacy in bone and soft tissue sarcoma has also been recognized (25–27). However, due to the low incidence of bone and soft tissue sarcoma, published studies are often single-center, retrospective, non-randomized, and non-controlled, combined with a limited sample size. Therefore, strong supportive evidence of clinical efficacy and safety is lacking for apatinib for the treatment of bone and soft tissue sarcoma.

## Materials and Methods

### Sources of Data and Search for Studies

Pubmed, Embase, Medline, Web of Science, ScienceDirect, Ovid, Cochrane library, Vip (China), CNKI (China), Wanfang (China), and CBM (China) databases were searched to identify all relevant articles up to September 1, 2020 in all languages. Additional relevant literature was also searched, including from the most important conferences (American Society of Clinical Oncology, European Society of Medical Oncology, and Chinese Society of Clinical Oncology) and dissertations with available data were also retrieved. Finally, the reference lists of articles selected for the present review were checked for additional articles.

The literature retrieval strategy combined two parts, adapted for each specific database: 1) diagnosis: “sarcoma” OR “osteosarcoma” OR “soft tissue sarcoma” OR “bone sarcoma”; 2) agent name: “apatinib” OR “apatinib mesylate” OR “YN968D1”. The two parts were combined with “AND” using Boolean logic.

### Study Selection

Studies fulfilling the following criteria were included: 1) Enrolled patients with histologically confirmed bone or soft tissue sarcoma; 2) Patients treated with oral apatinib at a daily dose of 250-750 mg; 3) Clinical efficiency outcomes were reported as tumor response, including complete response (CR), partial response (PR), stable disease (SD), and progressive disease (PD); 4) Adverse events (AEs) related to the treatment of all grades of tumor were reported.

Studies not adhering to the inclusion criteria were excluded, in addition to the following exclusion criteria: 1) Studies published elsewhere; 2) Animal experiments, laboratory research, reviews, meta-analyses or letters; 3) Articles with no accessible valid data; 4) Inclusion of fewer than 20 cases; 5) Participants received concomitant medication (e.g. chemotherapeutic agents) or surgery during the period of apatinib administration.

To avoid selection bias, particular studies were included or excluded where the two independent investigators agreed. A third reviewer resolved disagreements between them by discussion and formation of a consensus. The equations should be inserted in editable format from the equation editor.

### Evaluation of the Quality of Literature

The Methodological Index for Non-randomized Studies (MINORS) (28) was used to assess the quality of the literature included in the review, based on the following 12 items: stated aim of the study; inclusion of consecutive patients; prospective collection of data; an endpoint appropriate to the aim of the study; unbiased evaluation of endpoints; follow-up period appropriate to the major endpoint; loss to follow up not exceeding 5%; prospective calculation of sample size; control group having the gold standard intervention; contemporary groups; baseline equivalence of groups; and statistical analyses adapted to study design. Both non-comparative and comparative studies were assessed using the first 8 items, while only comparative studies were also evaluated using the remaining 4. The individual categories described above scored 0-2 points, so the quality score of an article ranged from 0 to 24 points.

### Data Extraction

Data were independently extracted by two investigators using a standardized Microsoft Excel spreadsheet, then fully reviewed by a third reviewer. The extracted information included: 1) basic information of article: first author, publication year, range of study years, and study type; 2) baseline characteristics of the enrolled patients: sample size, age, gender, histopathological diagnosis, disease state, and previous treatment; 3) apatinib administration: usage and dosage; 4) efficacy outcomes: complete response (CR), partial response (PR), stable disease (SD), progressive disease (PD), objective response rate (ORR, defined as CR+PR), disease control rate (DCR, defined CR+PR+SD), and progression-free survival rate (PFR); 5) incidence and grade of all AEs related to the administration of apatinib. Tables should be inserted at the end of the manuscript.

### Statistics Analysis

Dichotomous outcomes (CR, PR, SD, PD, ORR, DCR, PFR, and AEs) were reported as counts and proportions. Statistical analysis was conducted using the “meta” package of R statistical software (version 3.6.2, MathSoft, Massachusetts). Inter-study heterogeneity was evaluated using both Q tet and I2 tests, where p <0.1 and I2 >50% denoted heterogeneity. A fixed-effects model was used for pooled analysis where inter-study heterogeneity existed, otherwise, a random-effects model was adopted. Sensitivity analysis was conducted (where more than 9 studies were included) to evaluate the stability and credibility of the pooled results by exclusion of any single study from the meta-analysis one-by-one. Subgroup analysis was performed in terms of tumor type for bone sarcoma versus soft tissue sarcoma (STS). Additionally, Egger’s regression test was performed for the evaluation of publication bias and small study bias.

## Results

### Study Selection

**Figure 1:**
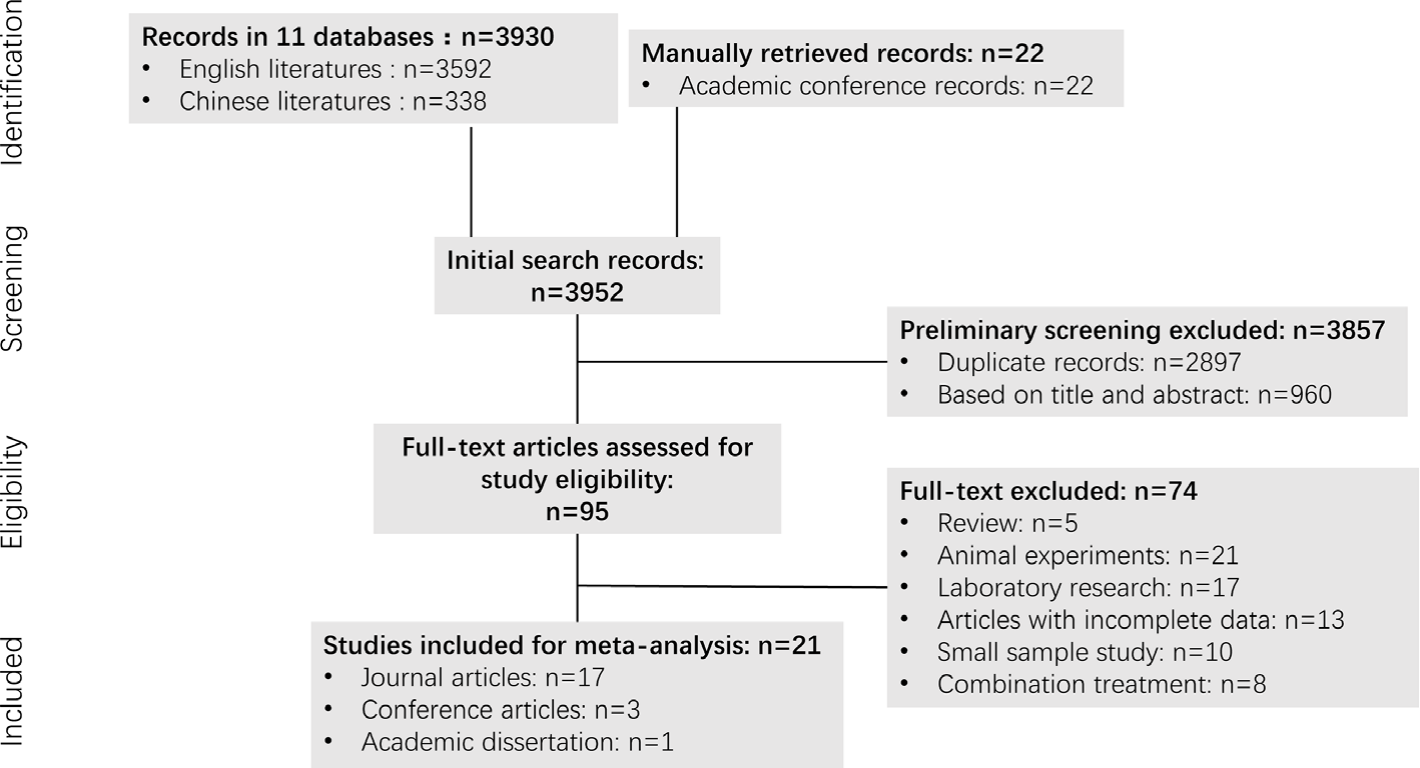
The flowchart of literature selection on meta-analysis of apatinib for bone and soft tissue sarcoma.

A flowchart of the study retrieval process is shown in **Figure 1**. A total of 3,952 potential studies were initially identified from the initial search of the aforementioned databases prior to screening the titles and abstracts, of which 2,897 were duplicates. After removal of the duplicates and screening of the titles and abstracts, a total of 95 of the initial records remained, the full-text documents of which were assessed, from which 74 were excluded. Those included: 1) Review articles (N=5); 2) Animal experiments (N=21); 3) Laboratory research (N=17); 4) Articles with incomplete data (N=13); 5) Studies with a small sample size (N=10); and 6) Combination treatment (N=8). Ultimately, 21 studies (29–49) were selected for the meta-analysis that included 827 patients, of which 17 were journal articles, 3 were conference articles and one was a dissertation.

### Main Characteristics of Selected Studies

The principal characteristics of the included articles are displayed in **Table 1**. The age of the enrolled patients ranged from 3 to 83 years with a gender ratio (female: male) of 1:1.47. Of the included studies, 7 investigated bone sarcoma patients, 5 studied STS patients, while the remaining 9 evaluated both. Apatinib was used in refractory bone sarcoma and STS, which possessed at least one of the following conditions: 1) distant metastasis (lungs or another organ); 2) postoperative recurrence; 3) a tumor that was unresectable; 4) failure of radiotherapy or chemotherapy. The initial dose of apatinib used in these studies was 425-750mg/d, except for children, for which an initial dose of 250 mg/d was used. The mean MINORS quality scores of the articles included in the analysis was 10.48 ± 1.75 (range: 7-13).

**Table 1:** The main characteristics of included studies.

Study ID	Study period	Sample Size	Gender	Age (range)	Diagnosis	Disease State	Treatment	Study Type	Quality Score
			(female/male)						
Li et al. (29)	2015.8-2017.3	23	NA	NA	Bone and soft tissue sarcomas	Recurrence; Metastasis; Radiotherapy and chemotherapy failure	500mg/d, po, qd	Journal articles	10
Yu et al. (30)	2015.7-2016.11	26	8/18	54.5	Osteosarcoma	Metastasis; Chemotherapy failure	500mg/d, po, qd or 250mg/d, po, bid	Conference articles	10
Zhou et al. (31)	2015.5-2016.11	34	NA	NA	Osteosarcoma	Advanced sarcoma; Lung metastasis (18 cases)	250mg/d (5cases); 425mg/d (3 cases); 500mg/d (26 cases); dosage adjustment (8 cases)	Conference articles	8
Yang et al. (32)	2015.9-2018.2	64	NA	NA	Bone and soft tissue sarcomas	Stage IV; Chemotherapy failure	500mg/d, po, qd	Conference articles	8
Zhu et al. (33)	2015.9-2016.8	31	16/15	49.00 (3-71)	Bone and soft tissue sarcoma	Stage IV; Metastasis; Relapse	425mg/d, po, qd; 9 for temporary discontinuation; 7 for reduction	Journal articles	10
Lv et al. (34)	2015.5-2017.5	28	10/18	54.5	Soft tissue sarcoma	Stage IV; Chemotherapy failure	500mg/d, po, qd	Journal articles	7
Xie et al. (35)	2015.6-2016.12	44	NA	NA	Bone and soft tissue sarcoma	Unresectable; Advanced sarcoma; Metastatic	750mg (BSA>1.5); 500mg (BSA<1.5); 250mg (age<10)	Journal articles	12
Kang (36)	2017.6-2018.9	21	9/12	38.3 (15-77)	Bone and soft tissue sarcoma	Advanced sarcoma; Unresectable; Relapsed; Metastatic	500mg/d, po, qd	Academic dissertation	11
Xie et al. (37)	2016.3-2017.7	37	11/26	23.4 (16-62)	Osteosarcoma	Unresectable; Metastatic Advanced sarcoma	750mg/d, po, qd for 31 cases, 500mg/d, po, qd for 6 cases	Journal articles	13
Zuo et al. (38)	2016.1-2018.5	31	17/14	15-71	Soft tissue sarcoma	Advanced sarcoma	500mg/d, po, qd, for 10 cases; 425mg/d, po, qd, for 19 cases; 250mg/d, po, qd, for 2 cases	Journal articles	9
Tian et al. (39)	2016.1-2017.8	27	11/16	22.22	Osteosarcoma	Chemotherapy failure; Unresectable lesions or distant metastasis	750mg/d, po, qd, for 11 cases; 500mg/d, po, qd, for 16 cases	Journal articles	11
Liao et al. (40)	2015.9-2018.2	64	31/33	42.16 (11-83)	Bone and soft tissue sarcoma	Stage IV; Chemotherapy failure	500mg/d, po, qd	Journal articles	11
Yao et al. (41)	2017.5-2018.7	45	16/29	43.02 (13-72)	Bone and soft tissue sarcoma	Chemotherapy failure; Unresectable lesions or distant metastasis	500mg/d, po, qd for 39 cases, 250 mg, po, qd for 6 cases	Journal articles	12
He et al. (42)	2016.9-2018.3	22	NA	NA	Bone and soft tissue sarcoma	Stage IV	500mg/d, po, qd	Journal articles	8
Gong et al. (43)	2015.1-2018.7	27	12/15	30.20 (8-71)	Soft tissue sarcoma	Unresectable lesions or distant metastasis	500mg/d, po, qd, for 26 adults; 250mg/d, po, qd, for 1 child	Journal articles	9
Liu et al. (44)	2015.3-2018.12	105	33/72	33.00 (18-58)	Osteosarcoma	Chemotherapy failure; metastasis	500mg/d, po, qd, for 57 patients; 750mg/d, po, qd, for 48 patients	Journal articles	11
Liu et al. (45)	2015.9-2018.2	42	23/19	47.67 (14-83)	Soft tissue sarcoma	Stage IV; Chemotherapy failure	500mg/d, po, qd	Journal articles	12
Wang et al. (46)	2016.3-2019.10	21	11/10	31.50 (8-71)	Synovial sarcoma	Chemotherapy failure	500mg/d, po, qd, for 20 adults; 250mg/d, po, qd, for 1 child	Journal articles	12
Tian et al. (47)	2016.5-2019.2	68	31/37	35.88	Bone and soft tissue sarcoma	Chemotherapy failure; Lung metastasis	500mg/d, po, qd	Journal articles	12
Xie et al. (48)	2009.10-2019.11	33	9/24	41.00 (17-72)	Chondrosarcoma	Unresectable	500mg/d, po, qd	Journal articles	13
Liao et al. (49)	2015.9-2019.12	34	11/23	35.24 (11-73)	Osteogenic sarcoma	Stage IV	500mg/d, po, qd	Journal articles	11

NA, not available; BSA, body surface area; po, peros (oral administration); qd, quaque die (once a day); bid, bis in die (twice a day).

### Clinical Efficacy Response

**Figure 2:**
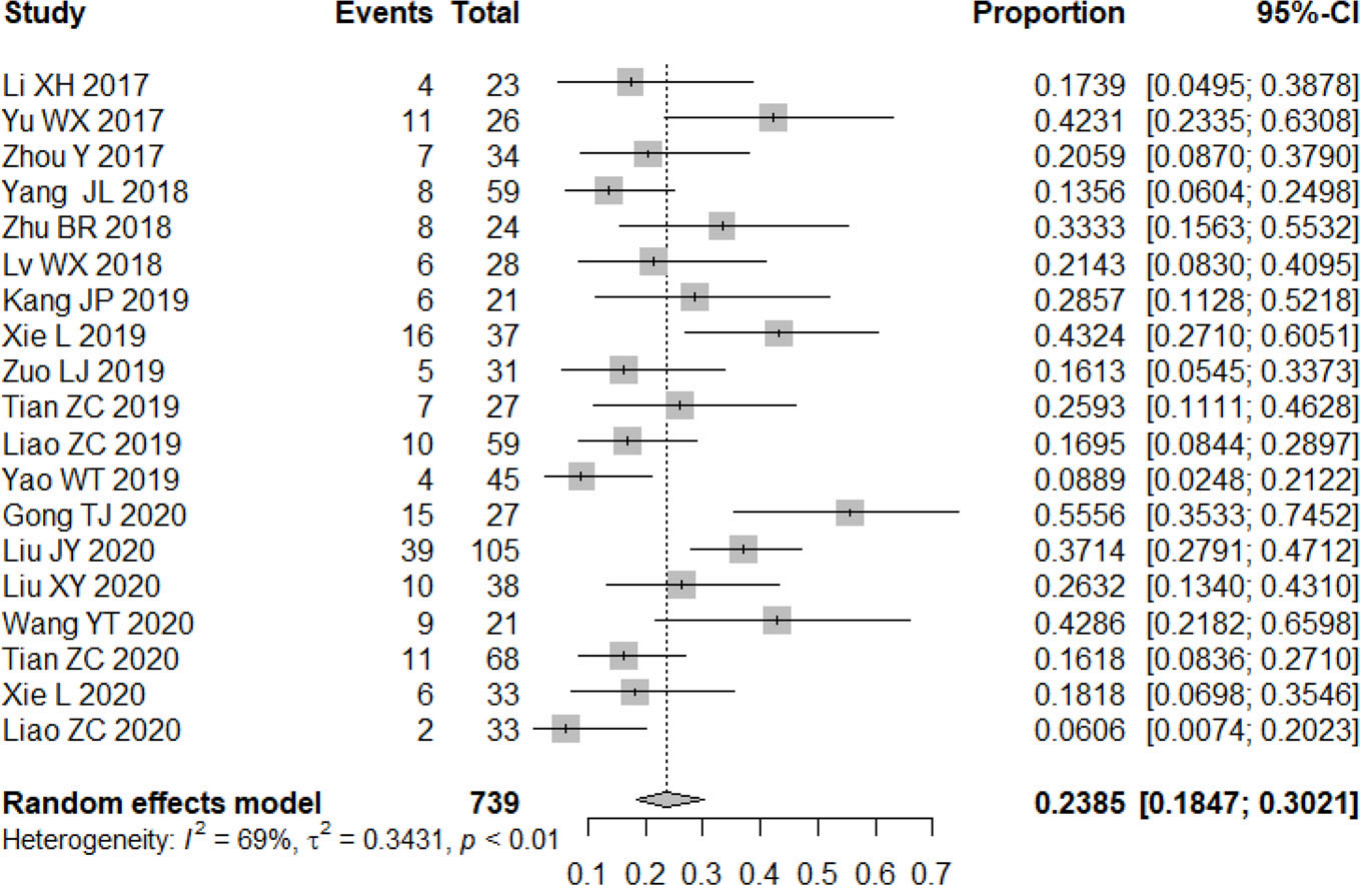
Forest plot showing the ORR of apatinib in the treatment of bone and soft tissue sarcomas.

**Figure 3:**
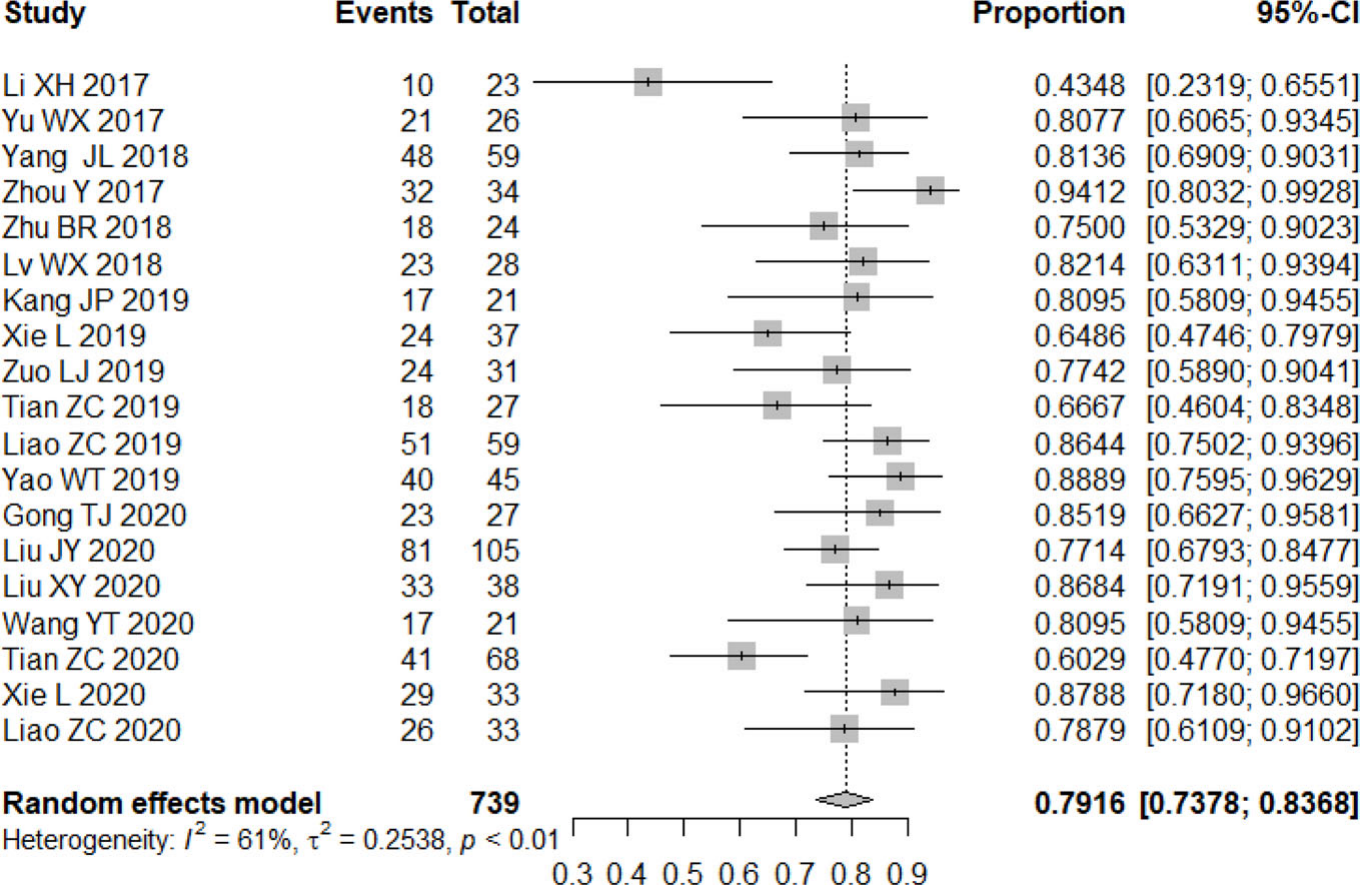
Forest plot showing the DCR of apatinib in the treatment of bone and soft tissue sarcomas.

All studies included in the analysis reported the efficacy response of apatinib for bone and soft tissue sarcoma. A random-effects or fixed-effects model was selected for the meta-analysis depending on the calculated heterogeneity of data in the statistical analysis. Pooled results indicated that the ORR of apatinib for the treatment of bone and soft tissue sarcoma was 23.85% (95% confidence interval (CI): 18.47%-30.21%; heterogeneity: I^2^ = 69%, p<0.01) (**Figure 2**), while DCR was 79.16% (95% CI: 73.78%-83.68%; heterogeneity: I^2^ = 61%, p<0.01) (**Figure 3**). Pooled CR, PR, SD, and PD rate were 0.04% (95% CI: 0.00%-0.64%), 24.20% (95% CI: 18.67%-30.74%), 52.71% (95% CI: 44.26%-61.00%), and 21.98% (95% CI: 17.30%-27.93%), respectively. In summary, the majority of patients benefited from treatment with apatinib where efficacy for PR or SD was considered.

### PFS

**Figure 4:**
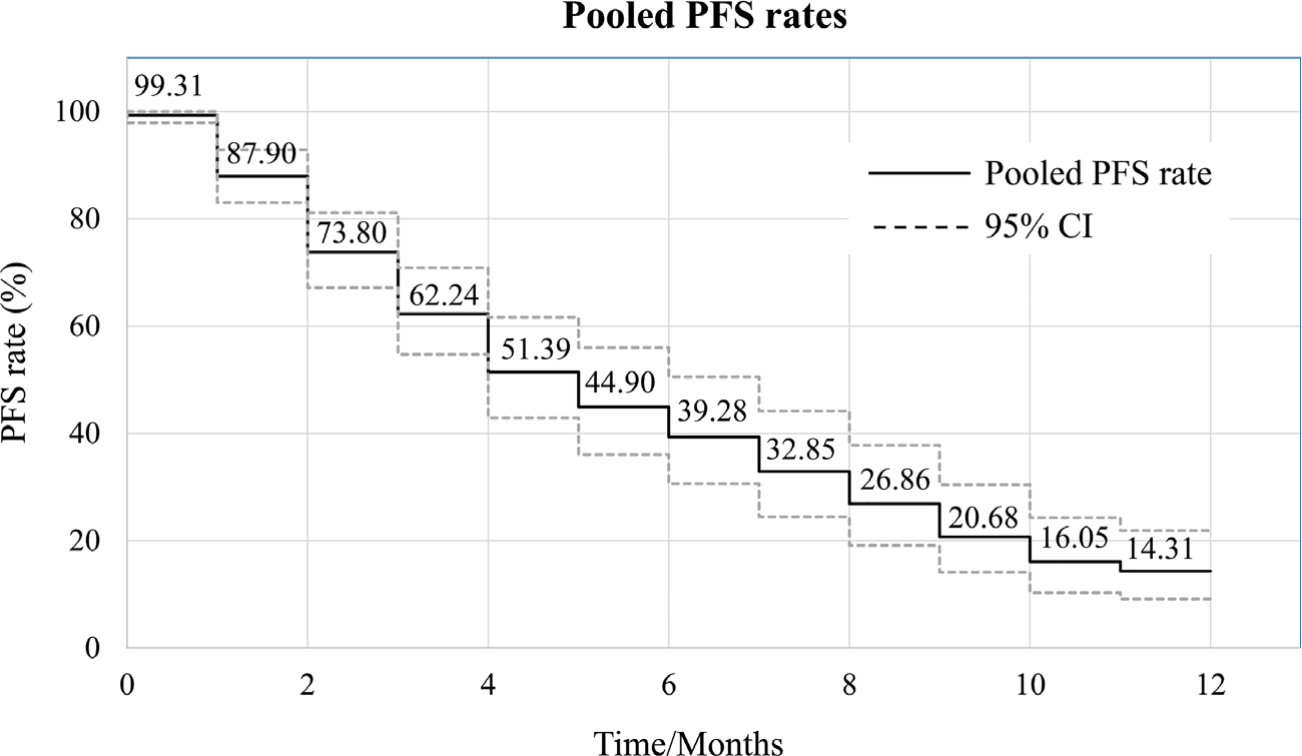
Pooled survival curve regarding progression-free survival (PFS) (each PFR was synthesized using meta-analysis of single rate).

Of the 21 studies included in the analysis, 15 reported PFS for all patients after administration of apatinib. Median PFS ranged from 3.5 to 13.1 months, with a mean of 7.08 ± 2.98 months. Pooled PFR at various time points was calculated using the single rate meta-analysis method, from which a meta-pooled PFS curve was generated (**Figure 4**). The results demonstrated that pooled PFRs in patients with bone and soft tissue sarcomas after 1, 6, and 12 months were 99.31% (95% CI: 97.87%-99.96%), 44.90% (95% CI: 36.03%-55.96%), and 14.31% (95% CI: 9.08% to 21.84%), respectively.

### Incidence of Adverse Events

Adverse events were reported in 19 studies, involving 756 patients. The incidence of adverse events was extracted to systematically evaluate the safety profile of apatinib treatment (**Table 2**). The severity of adverse events was graded using the National Institute-Common Terminology Criteria (CTCAE). The meta-analysis indicated that the three most commonly reported adverse events were hand-foot syndrome, hypertension, and fatigue, with an incidence of 41.13% (95% CI: 33.02%-49.49%), 36.15% (95% CI: 28.00%-44.73%), and 20.52% (95% CI: 13.14%-27.89%), respectively. Diarrhea, anorexia, and proteinuria were also relatively common, their incidence exceeding 10%. Furthermore, the incidence of grade 3-4 adverse events was significantly lower, of which hypertension (3.19%, 95% CI: 1.73%-4.64%) and hand-foot syndrome (2.71%, 95% CI: 1.36%-4.07%) were the two most common. It should be noted that none of the reported adverse events resulted in permanent morbidity, and they were alleviated by withdrawal, dose adjustment, or symptomatic treatment.

**Table 2:** The pooled incidence of adverse events.

Adverse event	Pooled Incidence (%)	95%CI	Heterogeneity		Publication bias (p-value)*
			I^2^ (%)	p-value	
**All grades**					
Hand-foot syndrome	41.13	33.02-49.49	81	<0.01	0.532
Hypertension	36.15	28.00-44.73	83	<0.01	0.466
Fatigue	20.52	13.14-27.89	90	<0.01	<0.01
Diarrhea	19.70	13.80-26.28	73	<0.01	0.179
Anorexia	16.16	10.22-22.10	90	<0.01	<0.01
Proteinuria	13.13	6.14-21.97	90	<0.01	0.175
Mucositis	6.23	2.36-11.42	80	<0.01	0.045
Rash	5.46	0.94-12.45	89	<0.01	0.615
Hair hypopigmentation	5.09	0.20-13.91	93	<0.01	0.855
Pneumothorax	5.05	1.71-9.60	78	<0.01	0.126
Weight loss	4.61	0.37-11.83	91	<0.01	0.119
Myelosuppression	4.42	1.24-9.42	86	<0.01	0.111
Pain	4.21	0.61-9.89	86	<0.01	<0.01
Wound healing	2.36	0.42-5.31	68	<0.01	0.185
Nausea	1.80	0.00-5.66	82	<0.01	0.043
Elevated bilirubin	1.01	0.01-2.95	52	<0.01	0.460
Elevated transaminase	0.82	0.01-1.97	45	0.02	0.201
Hypertriglyceridemia	0.61	0.00-2.81	70	<0.01	0.127
Thrombocytopenia	0.56	0.00-2.14	50	<0.01	0.133
Hematuresis	0.40	0.00-1.33	32	0.10	0.048
Coagulation disorder	0.13	0.00-0.86	34	0.08	0.032
Headache	0.08	0.00-0.73	14	0.29	0.021
Hoarseness	0.00	0.00-0.39	0	0.99	<0.01
**Grade 3-4**					
Hypertension	3.19	1.73-4.64	45	0.01	0.524
Hand-foot syndrome	2.71	1.36-4.07	38	0.05	0.162
Pneumothorax	1.38	0.17-3.30	52	<0.01	0.987
Diarrhea	1.32	0.43-2.53	0	0.62	0.451
Proteinuria	1.13	0.31-2.29	32	0.08	0.032
Anorexia	1.10	0.02-3.14	61	<0.01	0.963
Fatigue	0.93	0.19-2.03	0	0.68	0.009
Wound healing	0.66	0.00-2.46	61	<0.01	0.394
Rash	0.18	0.00-0.91	0	0.53	0.110
Thrombocytopenia	0.05	0.00-0.63	0	0.88	0.077
Leukocytopenia	0.02	0.00-0.50	0	0.93	0.068
Anemia	0.01	0.00-0.48	0	0.99	<0.01
Mucositis	0.00	0.00-0.45	0	1.00	0.125
Neutropenia	0.00	0.00-0.38	0	0.97	0.035
Coagulation disorder	0.00	0.00-0.32	0	1.00	<0.01

*Egger regression test for publication bias.

### Subgroup analysis (Bone Sarcoma vs. STS)

**Figure 5:**
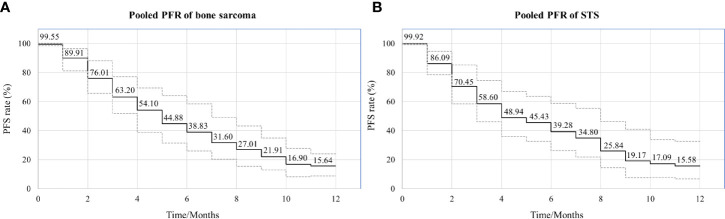
Pooled estimates of PFS between the two subgroups. **(A)** PFS curve in bone sarcoma; **(B)** PFS curve in STS.

Both bone sarcoma and STS were included in the present meta-analysis, differing in their histological origins. Subgroup analysis was performed based on the category of tumor (bone sarcoma vs. STS). Eleven articles referred specifically to bone sarcoma and 8 to STS in the 21 articles included in the meta-analysis. The results of subgroup analysis indicated that the ORR and DCR of apatinib for bone sarcoma were 22.62% (95% CI: 12.19%-33.04%) and 76.94% (95% CI: 68.58%-83.61%), respectively, while for STS they were 29.03% (95% CI: 20.53%-41.06%) and 79.94% (95% CI: 68.94%-89.02%), respectively. Similarly, comparable values for pooled PFS were obtained in the two subgroups, with 6-month rates of PFS of 44.88% (95% CI: 31.45%-64.06%) and 45.43% (95% CI: 32.52%-63.46%) for the bone sarcoma and STS groups, respectively, and 12-month rates of PFS of 15.64% (95% CI: 8.75%-23.87%) and 15.58% (95% CI: 6.62%-32.46%) (**Figure 5**). The incidence of adverse events was similar for the two subgroups. Details of the most common adverse events, such as hand-foot syndrome and hypertension, are listed in **Table 3**.

**Table 3:** The results of subgroup analysis (bone sarcoma vs. STS).

	Bone sarcoma					STS				
	N	Pooled rate (%)	95%CI	Heterogeneity		N	Pooled rate (%)	95%CI	Heterogeneity	
				I^2^ (%)	p-value				I^2^ (%)	p-value
**Efficacy response**										
CR	367	0.00	0.00-0.58	0	0.99	239	0.26	0.00-1.95	0	0.93
PR	367	21.60	12.62-32.04	78	<0.01	239	28.48	20.36-39.83	62	0.01
SD	367	51.40	37.49-65.09	83	<0.01	239	54.53	48.41-61.43	45	0.08
PD	367	25.11	17.76-35.52	71	<.0.01	239	20.10	10.92-31.01	71	<0.01
ORR	367	22.62	12.19-33.04	87	<0.01	239	29.03	20.53-41.06	66	<0.01
DCR	367	76.94	68.58-83.61	61	0.02	239	79.94	68.94-89.02	74	<0.01
**Adverse event**										
**All grades**										
HFS	315	42.17	20.41-65.56	94	<0.01		44.08	32.67-59.48	63	0.03
Hypertension	315	28.75	13.35-43.98	94	<0.01		35.99	15.99-58.71	87	<0.01
**Grade 3-4**										
HFS	315	5.01	1.36-8.66	51	0.05	188	4.27	1.46-8.07	48	0.09
Hypertension	315	2.79	0.67-4.90	49	0.06	188	4.23	0.00-8.89	58	0.04

CR, complete response; PR, partial response; SD, stable disease; PD, progressive disease; ORR, objective response rate; DCR, diease control rate; HFS, hand-foot syndrome.

### Sensitivity Analysis

Sensitivity analysis was evaluated to assess the stability of the results and explore potential sources of heterogeneity. By excluding studies one-by-one, the recalculated results indicated that no substantial change in pooled ORR (maximum deviation: 5.41%), pooled DCR (maximum deviation: 1.38%), or pooled rate of PFS (maximum deviation: 0.26%, 4.94%, and 9.36% for 1, 6, and 12 months, respectively) was observed. The reanalyzed pooled incidence of predominant adverse events (incidence >10%) deviated within a range of 5.79% to 13.94%. Notably, the lower the incidence, the greater the deviation of the results, possibly due to the small sample size effect, that is, the majority of the studies included in the pooled analysis exhibited an incidence of 0. From this, we deduce that the pooled results of this meta-analysis were stable and reliable (**Table 4**).

**Table 4:** Sensitivity analysis of the main pooled results.

	Pooled results (%)	Sensitivity analysis (%)		Maximum deviation (%)
		Min	Max
**Efficacy response rate**				
CR*	0.04	0.01	0.06	75.00
PR	24.20	22.97	25.59	5.74
SD	52.71	51.45	54.68	3.74
PD	21.98	20.92	22.82	4.82
ORR	23.85	22.56	25.12	5.41
DCR	79.16	78.08	80.25	1.38
**PFS rate**				
1-month	99.31	99.18	99.57	0.26
6-month	44.90	42.68	46.59	4.94
12-month	14.31	12.97	15.62	9.36
**The incidence of adverse events (All grades)**				
HFS	41.13	38.75	42.45	5.79
Hypertension	36.15	33.71	38.05	6.75
Fatigue	20.52	18.64	22.00	9.16
Diarrhea	19.70	18.30	20.49	7.11
Anorexia	16.16	14.67	17.60	9.22
Proteinuria	13.13	11.30	14.60	13.94

CR, complete response; PR, partial response; SD, stable disease; PD, progressive disease; ORR, objective response rate; DCR, diease control rate; HFS, hand-foot syndrome.

*An obvious deviation was detected because most of the included studies for pooled analysis had an incidence of 0.

### Publication Bias

**Figure 6:**
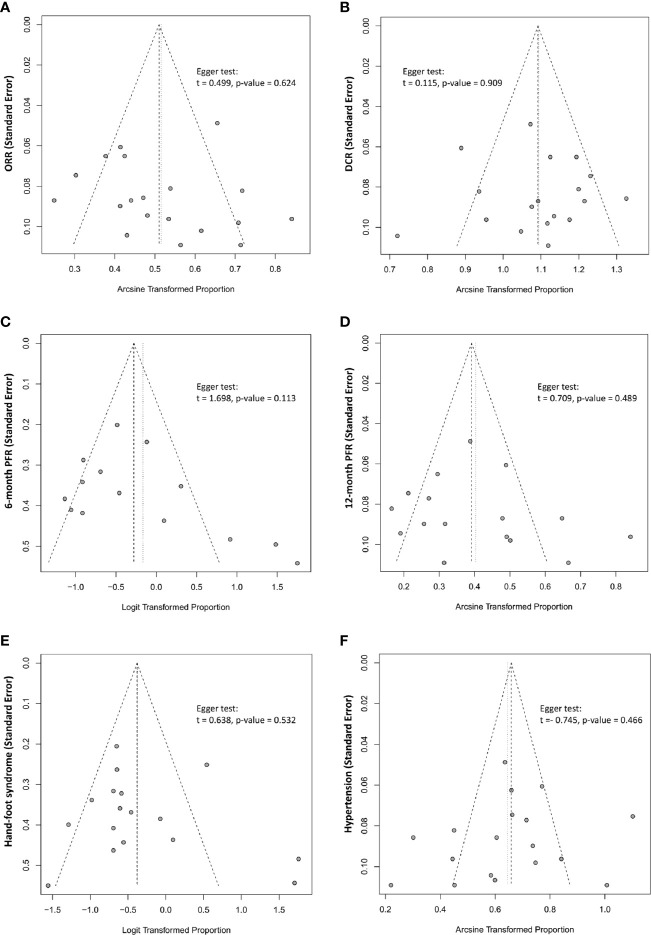
The funnel plot and Egger linear regression test assessing publication bias. **(A)** ORR (p=0.624); **(B)** DCR (p=0.909); **(C)** 6-month PFR (p=0.113); **(D)** 12-month PFR (0.489); **(E)** AE-Hand-foot syndrome (0.532); **(F)** AE-Hypertension (p=0.466).

Tests for publication bias were performed on the primary outcome measures (ORR, DCR, 6-month PFS rate, 12-month PFS rate, and incidence of adverse events) in the present meta-analysis. Publication bias was assessed using funnel plots and an Egger’s regression test, finding no publication bias in the present meta-analysis (**Figure 6**).

## Discussion

Despite the development of chemotherapeutic agents, surgical techniques, and multi-modal treatments, long-term survival remains stagnant and poor for patients with advanced, refractory, metastatic, or relapsed bone and soft tissue sarcoma. Specifically, median overall survival (OS) was found to be less than 11 months and 12.5 months for advanced bone sarcomas and advanced STS, respectively (5). The 5-year OS rate is less than 30% and 45%, respectively. Because of the recognition of the critical role of tyrosine kinase in tumor angiogenesis, studies have focused on the development of TKIs as target-specific treatments in anti-tumor therapy by interfering with the VEGF/VEGFR signaling pathway. At least 20 TKIs (sorafenib, sunitinib, cediranib, pazopanib, regorafenib, imatinib, etc.) have been approved as anti-tumor agents by the FDA, some exhibiting promising efficacy with complications that are tolerable for the treatment of advanced diseases, such as gastric cancer (GC), hepatocellular carcinoma (HCC), non-small-cell lung carcinoma (NSCLC), colorectal cancer (CRC), and ovarian cancer (OC), etc.

Multiple preclinical studies and clinical trials have indicated that apatinib may act as a novel and promising VEGFR2-TKI for use in the treatment of bone and soft tissue sarcoma (18, 22, 23, 27). Its antitumor properties include vascular normalization, tumor regression (50), reversal of multidrug resistance (18), tumor microenvironment (TME) optimization (51), and enhancement of antitumor immunity (52). There are inconsistencies in clinical outcomes and complications in the reported studies, however. Combining the clinical studies using a meta-analysis indicated that pooled ORR and DCR for the treatment of bone and soft tissue sarcoma using apatinib was 23.85% (95% CI: 18.47%-30.21%) and 79.16% (95% CI: 73.78%-83.68%), respectively. Such efficacy is comparable or even superior to other TKIs, including sorafenib, pazopanib, sunitinib, regorafenib, and anlotinib, although no direct comparative studies have yet been conducted (**Table 5**) (16, 53–70). It is noteworthy that pazopanib has been approved by the US FDA for the treatment of advanced STS as a second or third-line therapy. In a phase III clinical trial of pazopanib for advanced STS (PALETTE), the ORR and DCR were 5.69% (14/246) and 76.83% (189/246), respectively, but 10.57% (13/123) and 38.21% (47/123) in a real-world study (65). Therefore, for this comparison, apatinib represents among the most promising anti-angiogenic agents available for use as the preferable treatment option for advanced bone and soft tissue sarcoma.

**Table 5:** The main *anti*-*angiogenic* *TKIs for bone and soft tissue sarcoma*.

Compound	Targets	Refs	Histological subtypes	Sample size (n)	Clinical outcomes*			
					ORR	DCR	PFS (mo)	PFR-6
Apatinib	Mainly VEGFR2	Pooled results of the present meta-analysis	Multiple STSs	827	23.85%	79.16%	7.08 ± 2.98	44.90%
Sorafenib	VEGFR2, VEGFR3, PDGFR-α, PDGFR-β, c-kit, RET, RAF, FLT-3, FGFR-1	Chevreau et al. (53) Grignani et al. (16) von Mehren et al. (54) Pacey et al. (55) Santoro et al. (56)	EHE Osteosarcoma Multiple STSs Multiple STSs Multiple STSs	15 35 38 21 101	13.33% 14.29% 0% 14.29% 14.47%	84.62% 48.57% 37.84% 57.14% 47.36%	6.0 4.0 3.0 / 4.2	38.46% 17% 32% 0% 35%
Sunitinib	VEGFR1, VEGFR2, VEGFR3, PDGFR-α, PDGFR-β, c-kit, RET, FLT-3, FGFR-1	George et al. (57) Jo et al. (58)	Multiple STSs AF	48 19	2.08% 26.32%	22.92% 68.42%	1.8 /	14.5% /
Cediranib	VEGFR1, VEGFR2, PDGFR-α, PDGFR-β, c-kit, FLT-3	Kummar et al. (59) Judson et al. (60)	ASPS ASPS	46 32	34.88% 19.35%	95.35% /	/ 10.1	83.72% 59.38%
Pazopanib	VEGFR1, VEGFR2, VEGFR3, PDGFR-α, PDGFR-β, c-kit, FGFR-1	Martin-Broto et al. (61) Samuels et al. (62) Sleijfer et al. (63) Stacchiotti et al. (64) van der Graaf et al. (65)	SFT Liposarcoma Liposarcoma; Leiomyosarcoma; Synovialosarcoma; Others EMC Non-adipocytic STS	36 41 19; 42; 38; 43 26 246	6% 2% 0%; 2.38%; 13.16%; 6.98% 18.18% 5.69%	28.57% 43.9% / / / / 90.9% 72.36%	5.6 4.4 2.6; 3.0; 5.3; 3.0 19.4 4.6	/ 39% / / / / / 25.61%
Regorafenib	VEGFR1, VEGFR2, VEGFR3, PDGFR-β, c-kit, RET, RAF	Mir et al. (66) Duffaud et al. (67) Davis et al. (68)	Liposarcoma; leiomyosarcoma; Synovial sarcoma; Others Osteosarcoma Osteosarcoma	20; 28; 13; 28 26 22	0%; 0% 7.69%; 10.71% 7.69% 13.6%	45%; 85.71%; 84.61%; 75% 75% 65.38% /	1.1; 3.7; 5.6; 2.9 4.1 3.6	20%; 21%; 38%; 43% 34.62% 30%
Axitinib	VEGFR1, VEGFR2, VEGFR3, PDGFR-α, PDGFR-β, c-kit	Stacchiotti et al. (69)	SFT	17	5.9%	88.2%	9.4	M-SFT:69.2% D-SFT:25.0%
Anlotinib	VEGFR1, VEGFR2, VEGFR-3, PDGFR-α, PDGFR-β, c-kit, FGFR-1	Chi et al. (70)	Multiple STSs	166	12.65%	74%	5.6	30.72%

ORR, objective response rate; DCR, diease control rate; PFS, progression-free survival; PFR, PFS rate; EHE, epithelioid hemangioendothelioma; AF, Aggressive fibromatosis; ASPS, alveolar soft tissue part sarcoma; SFT, solitary fibrous tumour; EMC, extraskeletal myxoid chondrosarcoma; D-SFT, ‘dedifferentiated’ SFT; M-SFT, ‘malignant’ SFT.

*The clinical efficacy response was assessed based on RECIST criteria.

The current data indicate that the median PFS for patients that have received apatinib averaged 7.08 ± 2.98 mos (range: 3.5-13.1 mos), considered favorable in comparison with other TKI monotherapies (Sorafenib: 3.0-6.0 mos; Sunitinib: 1.8 mos; Pazopanib: 3.0-5.6 mos; Regorafenib: 1.1-5.6 mos; Anlotinib: 5.6 mos), although there is a lack of direct pairwise comparisons. Similarly, the pooled 6-month PFR (44.90%) for apatinib was also higher than other TKIs (17-43%) (**Table 5**). However, it should be noted that the pooled efficacy response of apatinib in the present meta-analysis was lower than that of other TKIs in specific histological sarcoma subtypes. In a phase II clinical trial of cediranib (30 mg) for patients with metastatic or nonresectable alveolar soft part sarcoma (ASPS), the efficacy response was encouraging, with the ORR and DCR found to be 34.88% and 95.35%, respectively, with a 6-month PFR of 83.72% (59). Furthermore, a number of studies also confirmed an enhanced anti-tumor response for ASPS, achieving a PFS in excess of 10 months (60). These inconsistencies in clinical results of different sarcoma subgroups may be partially due to tumor heterogeneity, especially tumor vascularity which has been shown to be closely associated with the effectiveness of anti-VEGFR therapy. Apatinib also achieved better clinical outcomes when treating vascular-rich sarcomas such as ASPS (71). To further explore the therapeutic efficacy of apatinib in distinct sarcoma subtypes, a subgroup meta-analysis was conducted for two separate subgroups: bone sarcoma and STS. Regrettably, no substantial difference was identified in the present analysis. Therefore, subsequent research strategies should focus on a single sarcoma and conduct head-to-head randomized controlled clinical trials to identify the influence of histological type on the effectiveness of antiangiogenic therapy.

In terms of toxicity, adverse events appear to be common in patients that have been administered apatinib, and are, to a certain extent, the most common reason for drug withdrawal. Hand-foot syndrome (41.13%), hypertension (36.15%), and fatigue (20.52%) are the three most common adverse reactions. Comparatively, the incidence of grade 3-4 adverse events was relatively low, with the most frequently reported events being hypertension (3.19%) and hand-foot syndrome (2.71%). The pooled drug-related toxicity obtained in this meta-analysis is consistent with previous experience of apatinib in other refractory malignancies, such as gastric cancer, non-small cell lung carcinoma, and breast cancer (20, 21, 23, 72). However, notably, all such toxicities reported in this study were controllable, and able to be reduced by the adjustment of dose, withdrawal or symptomatic treatment. It has been reported that the toxicity of apatinib is positively correlated with the dose and duration of medication. But this conclusion needs to be further verified for bone and soft tissue sarcoma.

We acknowledge that the study has limitations. Firstly, there was a lack of high-level evidence from randomized clinical trials that had a large number of patients, hence influencing the overall outcomes of the study. Additionally, there was considerable heterogeneity across the subtypes of bone and soft tissue sarcomas. Although subgroup analysis was conducted to better understand the differences in efficacy or safety of apatinib between bone sarcoma and STS, no positive results were obtained. Further research into specific histological subtypes of sarcomas is required for a complete analysis of tumor heterogeneity. Lastly, the study only included a review of apatinib monotherapy in clinical applications, however, apatinib combination therapy (e.g. apatinib plus chemotherapy, or apatinib plus targeted therapy) is also common, and their clinical efficacy and safety also require confirmation in additional clinical trials.

## Conclusion

Using the best evidence currently available, the results of the present meta-analysis indicate that apatinib has a beneficial influence in bone and soft tissue sarcoma, with favorable ORR, DCR, PFS, and PFR. Manifestations of toxicity are common during the administration of apatinib, although the majority are grade 1-2 and largely manageable by clinical intervention. Apatinib may thus benefit sarcoma patients, with favorable efficacy and an acceptable safety profile, although further high-quality clinical studies are required to further define its activity and toxicity in specific single subtype sarcoma.

## Data Availability Statement

The original contributions presented in the study are included in the article/supplementary material. Further inquiries can be directed to the corresponding authors.

## Author Contributions

ZL: Literature retrieval, data extraction, and article writing. MH and KL: Literature retrieval, data extraction, and literature quality evaluation. ML and JL: Data verification and literature quality evaluation. HZ: Statistical analysis. ZW: Study design and quality supervision. YL: Study design, statistical analysis, and manuscript modification. All authors contributed to the article and approved the submitted version.

## Conflict of Interest

The authors declare that the research was conducted in the absence of any commercial or financial relationships that could be construed as a potential conflict of interest.
